# Retraction: The seasonal reproduction number of dengue fever: impacts of climate on transmission

**DOI:** 10.7717/peerj.1069/retraction

**Published:** 2016-10-04

**Authors:** Sittisede Polwiang

**Affiliations:** Department of Mathematics, Faculty of Science, Silpakorn University, Thailand

Retraction: Polwiang S. (2015) The seasonal reproduction number of dengue fever: impacts of climate on transmission. PeerJ 3:e1069 https://doi.org/10.7717/peerj.1069

The author wishes to Retract this article. Some of the original data were supplied by the Thailand Bureau of Vector-Borne Disease, however at that time an incorrect assumption was made by the author when converting weekly data into monthly data. As a result, the number of 226 cases per 100,000 which is used in the model is incorrect. In addition, the figure of 0.5 used for the "infection rate in mosquito’s egg" (γ in equations 4a and 4b) is also incorrect (the author believes the figure should have been 0.15). These errors mean that many of the specific numerical results in the paper are in error, although the author believes that the overall conclusions are still correct and that the general shape of the graphs remains unchanged (albeit with different values). Because of these errors and the fact that the correct values for the input data are uncertain, the article is Retracted. Interested readers should note that the author has used the current best estimates for the correct data to update a preprint of this paper at https://peerj.com/preprints/756v5/.

